# Dysregulation of peripheral expression of the YWHA genes during conversion to psychosis

**DOI:** 10.1038/s41598-020-66901-1

**Published:** 2020-06-17

**Authors:** Fanny Demars, Oussama Kebir, Aude Marzo, Anton Iftimovici, Catherine Schramm, ICAAR Study Group, Isabelle Amado, Julie Bourgin, Claire Daban Huard, Célia Jantac Mam-Lam-Fook, Marion Plaze, Fabrice Rivollier, Marie-Odile Krebs, Boris Chaumette

**Affiliations:** 1 Université de Paris, Institut of Psychiatry and Neuroscience of Paris (IPNP), INSERM U1266, team Physiopathologie des Maladies Psychiatriques, GDR3557-Institut de Psychiatrie, Paris, France; 2 GHU Paris Psychiatrie et Neurosciences, Paris, France; 3 NeuroSpin, Commissariat à l’Energie Atomique, Gif-sur-Yvette, France; 4 University of Montreal, Sainte Justine Hospital Research Center, Montreal, Canada; 5 Department of Psychiatry, McGill University, Montreal, Canada

**Keywords:** Psychosis, Gene expression

## Abstract

The seven human 14-3-3 proteins are encoded by the YWHA-gene family. They are expressed in the brain where they play multiple roles including the modulation of synaptic plasticity and neuronal development. Previous studies have provided arguments for their involvement in schizophrenia, but their role during disease onset is unknown. We explored the peripheral-blood expression level of the seven YWHA genes in 92 young individuals at ultra-high risk for psychosis (UHR). During the study, 36 participants converted to psychosis (converters) while 56 did not (non-converters). YWHA genes expression was evaluated at baseline and after a mean follow-up of 10.3 months using multiplex quantitative PCR. Compared with non-converters, the converters had a significantly higher baseline expression levels for 5 YWHA family genes, and significantly different longitudinal changes in the expression of YWHAE, YWHAG, YWHAH, YWHAS and YWAHZ. A principal-component analysis also indicated that the YWHA expression was significantly different between converters and non-converters suggesting a dysregulation of the YWHA co-expression network. Although these results were obtained from peripheral blood which indirectly reflects brain chemistry, they indicate that this gene family may play a role in psychosis onset, opening the way to the identification of prognostic biomarkers or new drug targets.

## Introduction

Schizophrenia is a frequent disabling psychotic disorder, resulting from complex interactions between genetic factors and pre/post-natal exposure to environmental insults. It is a progressive illness that typically emerges during late adolescence and is characterized by several stages: early vulnerability, at-risk mental state (also called ultra-high risk, UHR), first episode of psychosis (FEP), and chronic disease^1^. It has been estimated that one third of UHR individuals convert to psychosis after three years of follow-up (converters), while a large majority remain with subthreshold symptoms, exhibiting a decrease in intensity or remission (non-converters)^2^. The biological mechanisms underlying this differential prognosis are not yet understood. We have previously reported that some methylomic and transcriptomic changes accompany the emergence of psychosis during adolescence^3,4^. However, additional longitudinal biological studies on UHR patients are crucially needed to identify biomarkers and to improve the current understanding of the pathophysiological mechanisms that accompany conversion from prodromes to major psychosis.

In recent decades, the importance of the YWHA gene family in neuropsychiatric disorders, including schizophrenia has been highlighted^5,6^. The YWHA genes (*YWHAB/G/E/Z/H/Q/S*) encode the 14-3-3 proteins (β, γ, ε, ζ, η, θ and σ, respectively), a family of highly conserved, multifunctional isoforms highly expressed in the brain^7,8^. YWHAS is also known as SFN, the gene coding for Stratifin. These proteins form homo- and heterodimers which bind other proteins among several hundreds of possible targets^9–11^. The YWHA gene family plays important roles in neuronal and synaptic development, function and plasticity^5,12–15^. In particular, they participate in the activation of the tyrosine and tryptophan hydroxylases, the rate limiting enzymes in the synthesis of some neurotransmitters including serotonin and dopamine^16^. Consistent with this latter role, we recently reported a low level of dopaminergic metabolites in the cerebrospinal fluid of a 20-year-old woman suffering from schizophrenia and carrying a *de novo* copy number variant (CNV; dup17p13.3) encompassing the *YWHAE* gene^17^. Moreover, genetic studies have reported multiple single nucleotide polymorphisms (SNPs) in *YWHAE*, *YWHAH* and *YWHAZ* in different cohorts of patients with schizophrenia^18–23^. Several transcriptomic and proteomic analyses have identified alterations in 14-3-3 protein isoforms in postmortem brain tissue of people with schizophrenia^6,24–26^. All these studies support the implication of the YWHA genes in chronic stages of schizophrenia. To our knowledge, only one study focused on early stages of this disease by exploring the levels of mRNA of the YWHA gene family in FEP^27^. This study reported a significantly lower expression of *YWHAB*, *YWHAE*, *YWHAG*, *YWHAQ* and a significantly higher expression of *YHWAS*. The authors proposed that the expression levels of the YWHA gene family could be used as indicators of schizophrenia severity.

This promising finding urged us to investigate whether the expression of the YWHA gene family is dysregulated in earlier stages of the disease, before the first-episode of psychosis, and during the emergence of psychotic symptoms. Therefore, we have used the longitudinal clinical and biological data of the French ICAAR cohort of psychiatric help-seeking young adults to explore the expression levels of the seven YWHA genes in UHR participants using multiplex quantitative-PCR (Q-PCR). We compared the baseline as well as the longitudinal changes of the expression levels between converters and non-converters.

## Results

### Demographic and clinical data of the study population

In total, 92 UHR participants were evaluated. Among them, 36 participants developed psychotic disorders during the follow-up (converters) and 56 did not (non-converters). The demographic and clinical characteristics (age, sex ratio, cannabis and tobacco use, psychotropic treatment intake, duration of the follow-up, severity of the symptoms, and RNA-sample quality) are reported in Table 1. At study start, there were no significant differences between converter and non-converter groups for daily use of tobacco, cannabis use within the last month, and psychotropic medication. There were no significant differences between groups in RNA quality from the samples obtained at the beginning and the end of the follow-up. The follow-up was longer for non-converters (p = 0.002), because converters were reassessed rapidly after conversion, whereas the follow-up was extended for non-converters to have greater confidence in concluding that outcome. As expected, the scores for clinical grading (BPRS, CGI) were not different at baseline but were higher in converters than non-convertors after the follow-up. Converters were younger and more frequently male than non-converters; consequently, the statistical models were adjusted with respect to age and sex.

**Table 1:** Demographic and clinical characteristics of the population.

Variable	Converters (N = 36)	Non-converters (N = 56)	P-value (Mann-Whitney or Fisher test)
Age	20.0 (2.53)	21.8 (3.65)	**0.023**
Sex ratio (male/female)	27/9	30/26	**0.049**
Tobacco (daily use)	50%	35.4%	0.25
Cannabis (use in the last 30 days)	37.5%	20.5%	0.12
Psychotropic medication at inclusion	62%	60%	1
Follow-up duration in months	9.8 (5.7)	13.7 (4.0)	**0.002**
BPRS at baseline	56.03 (13.88)	54.79 (12.11)	0.93
BPRS at the end of the follow-up	52.54 (14.00)	44.45 (11.36)	**0.016**
CGI at baseline	4.53 (0.84)	4.59 (0.82)	0.7
CGI at the end of the follow-up	4.78 (0.85)	3.74 (1.20)	**<10**^**−3**^
Quality of RNA measured by RIN	8.51 (0.45)	8.38 (0.54)	0.11

BPRS (Brief Psychiatric Rating Scale); CGI (Clinical Global Impressions Scale). P-values <0.05 are in bold. Values represent population mean (and standard deviation).

### Comparison of baseline expression of YWHA genes between converters and non-converters

**Figure 1:**
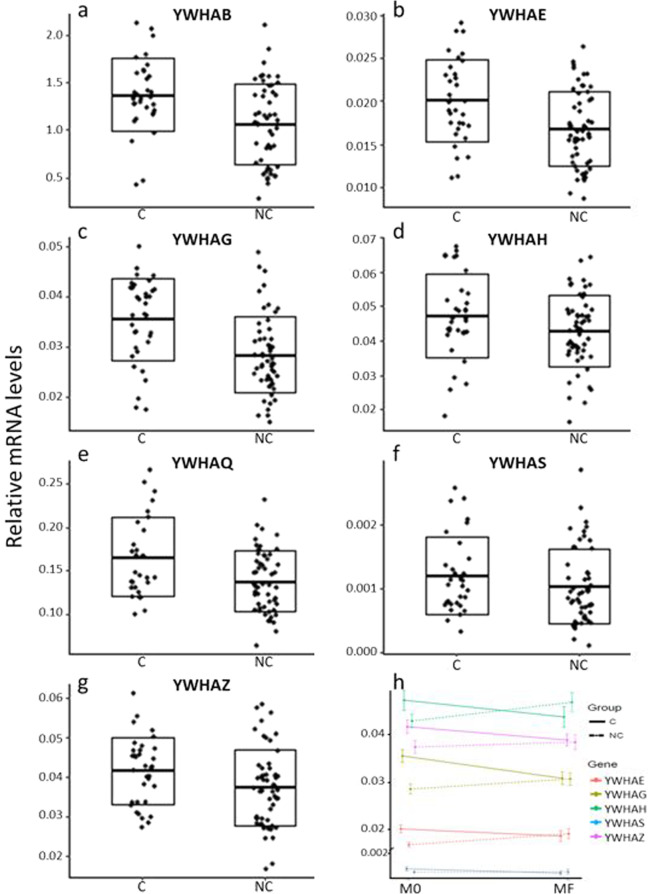
mRNA expression levels of the YWHA genes in converters and non-converters. (**a–g**) Baseline mRNA expression levels for YWHAB (**a**), YWHAE (**b**), YWHAG (**c**), YWHAH (**d**), YWHAQ (**e**), YWHAS (**f**), YWHAZ (**g**) in converters (C) and non-converters (NC). Each box plot represents the mean (central horizontal line) and the standard deviation. (**h**) Longitudinal changes between baseline (M0) and the end of the follow-up (MF) of mRNA expression of the five YWHA genes that are significantly different between converters (full lines) and non-converters (dashed lines). The error bars indicate the standard error to the mean.

In the logistic-regression analysis, the baseline expression levels for *YHWAB*, *YWHAE*, *YWHAG*, *YWHAQ* and *YWHAZ* were significantly higher in converters than in non-converters (Table 2, Fig. 1a–g, Supplementary Fig. 1). By contrast, the baseline expression levels of *YWHAH* and *YWHAS* were not significantly different between converters and non-converters. The baseline expression levels of *YWHAB*, *YWHAE*, *YWHAQ* and *YWHAS* were significantly correlated with several clinical scores at the end of the follow-up, especially with the score of negative symptoms (Supplementary Table 1).

**Table 2:** Comparison of baseline expression of YWHA genes between converters and non-converters using logistic regressions.

Gene	OR	95% Confidence Interval of OR	Non-parametric p-value	FDR-adjusted p-value
YWHAB	2.21	1.27: 3.86	0.003	**0.007**
YWHAE	2.24	1.32: 3.79	0.002	**0.007**
YWHAG	2.54	1.53: 4.21	0.0002	**0.0014**
YWHAH	1.55	0.98: 2.46	0.06	0.07
YWHAQ	1.84	1.13: 3.01	0.01	**0.014**
YWHAS	1.23	0.77: 1.96	0.41	0.41
YWHAZ	1.99	1.16: 3.41	0.009	**0.014**

OR represents the odd ratio. P-values <0.05 after 10 000 random permutations and FDR correction are in bold.

### Comparison of longitudinal changes in expression of YWHA genes between converters and non-converters

YWHA mRNA expression data at the end of the study were obtained for 67 patients (29 converters and 38 non-converters). We performed logistic-regression analyses of the longitudinal changes of YWHA expression levels between baseline and the end of the follow-up. Longitudinal changes of five of the seven YWHA genes (*YWHAE*,*YWHAG, YWHAH, YWHAS* and *YWAHZ*) were significantly different between non-converters and converters, with a decrease in their expression levels in converters (Table 3, Fig. 1h, Supplementary Fig. 2).

**Table 3:** Comparison of longitudinal changes in expression of YWHA genes between converters and non-converters using logistic regressions.

Gene	OR	95% Confidence Interval of OR	Non-parametric p-value	FDR-adjusted p-value
YWHAB	0.84	0.41: 1.71	0.65	0.65
YWHAE	−0.87	−1.60: -0.14	0.017	**0.028**
YWHAG	0.19	0.06: 0.63	0.004	**0.018**
YWHAH	0.39	0.17: 0.89	0.02	**0.028**
YWHAQ	0.54	0.28: 1.07	0.07	0.082
YWHAS	0.28	0.09: 0.81	0.02	**0.028**
YWHAZ	0.19	0.06: 0.63	0.005	**0.018**

OR represents the odd ratio. P-values < 0.05 after 10 000 random permutations and FDR correction are in bold.

### Comparison of the co-expression of YWHA genes between converters and non-converters

**Figure 2:**
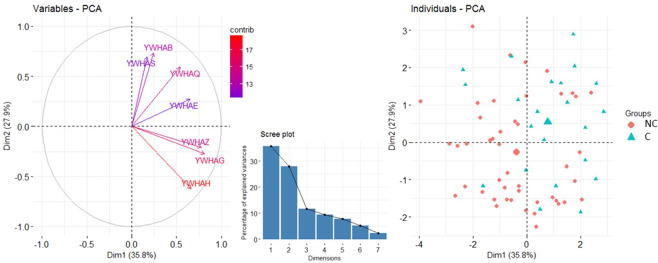
Principal Component Analysis of the expression levels of the YWHA genes at baseline. The Variables-PCA graph (left) displays the variable correlation plots of the principal component analysis with the respective contribution of each genes’ expression level indicated with a color gradient (contrib). The scree plot (center) indicates the percentage of the variance explained by each dimension. Dimensions 1 and 2 explain 35.8% and 27.9% of the variance respectively. The Individuals-PCA graph (right) displays the first two components values obtained for each participant. Values for converters (C) and non-converters (NC) are represented by blue triangles and red circles, respectively. The mean value for each group is represented by a larger symbol.

The members of the 14-3-3 family are known to form both homo- and heterodimers^9^. Therefore, we tested whether the mRNA expression levels of the different *YWHA* genes were correlated. We found that they mostly positively correlated with each other (Supplementary Table 2, Supplementary Fig. 3), suggesting that the expression of these genes was coordinated. To explore whether the YWHA mRNA expression network is dysregulated in the converters, we performed a principal component analysis (PCA) on the expression levels of YWHA genes at baseline (Fig. 2). More than 63% of the total variance was explained by the first two principal components (35.8% and 27.9%, respectively). The mRNA expression of *YWHAE*, *YWHAG*, *YWHAH*, and *YWHAZ* contributed highly to the variation captured in the first dimension (respectively 17%, 26%, 18% and 24%), and mRNA expression of *YWHAB*, *YHWAH*, *YWHAS* and *YWHAQ* contributed highly to the variation captured in the second dimension (respectively 27%, 20%, 25% and 18%). The values of the first principal component were significantly different between converters and non-converters, based on a logistic regression model of the first three principal components, with age, sex and medication as covariates (OR = 0.56, 95% CI = [0.37; 0.84], p-value after 10 000 random permutations = 0.004, Supplementary Fig. 1). In addition, the values of the first principal component significantly correlated with the at-risk clinical scores measured at the end of the follow-up by the CAARMS (Supplementary Table 1).

### Correlation between the methylation and expression levels of the YWHA genes

We investigated whether methylation levels on CpGs located in the promoters or the body of the YWHA genes were associated with their expression levels. We found significant correlations for *YWHAG* and *YWHAZ*, suggesting that the expression of these genes is influenced by the degree of methylation of their related DNA sequences. The correlation was positive between the expression of *YWHAG* and the level of methylation of 5 CpGs located in its coding sequence while the correlation was negative for the expression of *YWHAZ* and the level of methylation in a CpG located in its promoter (Supplementary Table 3).

## Discussion

In the present study, we assessed the mRNA expression of YWHA genes in a cohort of 92 UHR participants, of which 36 developed psychotic disorders in a follow-up period of up to 1 year. Longitudinal expression data were available for 67 participants. In converters, baseline mRNA levels of *YHWAB*, *YWHAE*, *YWHAG*, *YWHAQ* and *YWHAZ* were significantly higher than those in non-converters. Moreover, the longitudinal changes in the expression of *YWHAE*, *YWHAG, YWHAH, YWHAS* and *YWAHZ* were significantly different between converters and non-converters. The peripheral expressions of the YWHA genes were correlated and the values of the first principal component obtained for each subject by PCA were significantly different between converters and non-converters. In all participants, the values of the first principal component of the PCA and mRNA levels of *YWHAB*, *YWHAE* and *YWHAQ* at baseline were positively correlated with certain clinical symptoms at the end of the follow-up period. We observed significant correlations between the methylation and the expression levels of *YWHAG* and *YWHAZ*.

These results provide new insight on the implication of 14-3-3 proteins in schizophrenia. The comparison with other studies is difficult because, unlike the present study where peripheral blood was sampled, most of those studies have focused on expression in the post-mortem brain. However, in line with our results, higher levels of expression of *YWHAB*, *YWHAE*, *YWHAG* and *YWHAZ* have been described in the brains of schizophrenia patients compared with controls^28–31^. Yet, lower mRNA and protein expression of those genes in post-mortem brains of schizophrenia patients compared with controls has also been reported^24^. In the only study on early phases of psychosis, the mRNA expression level in peripheral-blood of *YWHAS* was higher in patients with first-episode psychosis (FEP) than in controls, whereas the levels of *YWHAB*, *YWHAE*, *YWHAG*, *YWHAQ* were lower^27^. They also reported reduced levels of the 14-3-3 proteins encoded by *YWHAB*, *YWHAE*, *YWHAG*, *YWHAQ* and *YWHAZ* in the blood of FEP patients. This apparent discrepancy with our study may come from the stages of the disease because the UHR stage precedes the FEP stage. The longitudinal decrease we observed during the conversion to psychosis brought the expression levels in converters closer to the levels described previously in FEP patients.

Our results suggest that a dysregulation of the expression of the YWHA gene family could be involved in the early pathological processes that lead to psychosis. Although the possible mechanisms involving 14-3-3 proteins in psychiatric disorders pathophysiology remain unknown, early overexpression of *YWHAE* and *YWHAG* has been shown to impair neurodevelopment, such as neuronal migration and neurite formation in animal models^32,33^. The higher expression levels observed at baseline in converters might therefore be a marker of impaired neurodevelopment. Several modifications in the cellular environment, such as inflammation or oxidative stress, affect the expression and the functions of the 14-3-3 proteins^34–38^. Interestingly, in the same cohort, we have shown that changes in DNA methylation occurs during the conversion to psychosis in genes involved in oxidative stress regulation and inflammatory pathways^4^. Therefore, the longitudinal decrease of the expression of five YWHA genes in converters could be related to the increased inflammatory and oxidative stress reported during the early phases of psychosis^39–42^.

The onset of psychosis is underpinned by complex molecular interactions between genetic vulnerabilities and environmental exposure leading to dynamic changes in gene expression. Our PCA analysis revealed a dysregulation of the co-expression network in converters compared with non-converters. This suggests a shared regulation possibly though some epigenetic mechanisms. For instance, we observed significant correlations between the methylation and the expression levels of *YWHAG* and *YWHAZ* in this cohort. Other epigenetic mechanisms like microRNA expression might also have play a role^43–48^.

Although there is evidence supporting the involvement on 14-3-3 in the pathophysiology of schizophrenia, some confounding factors have been proposed to explain changes in gene expression. Psychotropic medication appears to disrupt the expression of YWHA transcripts and 14-3-3 proteins in schizophrenia^31,49^. However, in the present study, there was no significant difference in treatment intake between converters and non-converters and antipsychotic doses were included in the regression analyses. Another concern is the tissue specificity of gene expression. Here, we measured gene expression in peripheral blood and not in the brain. However, the blood-brain correlation in gene expression has been reported to range from 0.25 to 0.64 and to be greater for genes highly expressed in both tissues^50^, as is the case for the YWHA family. The advantage of using blood samples is that it allows for longitudinal studies that can better inform us on the mechanisms of disease progression than transversal studies. Then, some individuals might have converted after the end of the follow-up; however, this does not interfere with the result about the longitudinal changes in YWHA genes expression because they have been sampled before this potential conversion.

Our study supports the implication of 14-3-3 proteins in the pathophysiology of schizophrenia onset. The measurement of 14-3-3 proteins in the cerebrospinal fluid has already been developed in neurology as a potential biomarker for neurodegenerative disorders, including Alzheimer’s disease, Parkinson’s disease, Creutzfeldt-Jakob disease, or amyotrophic lateral sclerosis^6,51,52^. Moreover, the 14-3-3 signaling pathway has emerged as a potential therapeutic target in neurology and could also represent a new opportunity in psychiatry^53,54^. Additional studies on larger cohorts of patients are needed to replicate and to further investigate the implication of this pathway in the pathophysiology of psychotic disorders.

## Methods

### Population

Participants were recruited in the French ICAAR cohort (PHRC AOM-07-118, promoted by Hôpital Sainte-Anne). The use of the cohort was approved by the institutional ethics committee “Comité de protection des personnes, Ile-de-France III, Paris, France” and written informed consent was obtained from all participants or their legal representatives if under age of 18, in accordance with the Declaration of Helsinki. The ICAAR cohort^55^ included 16- to-30-year-old help-seeking individuals, who had been consequently referred to the Adolescent and Young Adult Assessment Centre (Service Hospitalo-Universitaire, Hôpital Sainte-Anne, Paris, France) between 2009 and 2014. Inclusion criteria were alterations in global functioning (Social and Occupational Functioning Assessment Scale score <70) during the past year that were associated with psychiatric symptoms and/or subjective cognitive complaints. All help-seeking individuals were examined with the Comprehensive Assessment of At-risk Mental State protocol (CAARMS^56^), in its French translated version^57^ by specifically trained psychiatrists followed by a consensus meeting for best estimate diagnoses. Individuals meeting the CAARMS criteria for UHR status were included in the study. Exclusion criteria included conspicuous symptoms of psychosis, pervasive developmental disorder, bipolar disorder, or other established diagnoses, such as obsessive-compulsive disorder, severe or non-stabilized somatic and neurological disorders, head injury and an IQ score below 70. Participants were followed for one year at most and follow-up stopped either after a year or after conversion to psychosis. Participants underwent clinical assessment and blood sampling at two time points: at baseline (M0) and at the end of follow-up (MF). This design enabled intra-subject analyses, i.e., to detect changes occurring in gene expression between MF and M0, covering conversion to psychosis. The conversion to psychosis was determined using the CAARMS threshold (i.e., supra-threshold psychotic symptoms — thought content, perceptual abnormalities and/or disorganized speech — present for more than one week). UHR participants who reached the threshold during follow-up were considered converters while UHR participants who recovered or displayed persistent sub-threshold symptoms were considered non-converters. Each subject was assessed using several clinical scales: SOFAS, BPRS, PANSS (including the three subscales for positive symptoms, negative symptoms and disorganization), MADRS and YMRS. The CAARMS score was operationalized as the summed scores of the product of global rating scale score (0-6) and frequency (0–6) of the four first subscales as reported previously^58^. Levels of the current antipsychotic treatments were transformed into chlorpromazine equivalent doses that were used as a covariate in the regression analysis. References for the computation of chlorpromazine equivalent doses are available in the Supplementary Table 4.

### Q-PCR quantification

Total RNA was extracted from blood samples (PAXgene tubes) using a standard protocol with a QIAcube robot and PAXgene Blood RNA kit (QIAGEN). Quality control was done using LabChip GX (Perkin Elmer, Waltham USA). Complementary DNA (cDNA) synthesis was performed using Reverse Transcription Master Mix from Fluidigm according to the manufacturer’s protocol, with random primers in a final volume of 5 μL containing 100 ng total RNA, and incubation in a Nexus thermocycler (Eppendorf). cDNA samples were diluted by adding 20 μL of low TE buffer [10 mM Tris; 0.1 mM EDTA; pH = 8.0 (TEKNOVA)]. TaqMan probes were selected for each gene. For specific target pre-amplification, 1.25 μL of each diluted cDNA was used for multiplex pre-amplification with Fluidigm PreAmp Master Mix at 12 cycles. In a total volume of 5 μL, the reaction contained 1 μL of pre-amplification master mix, 1 μL of PCR water, 1.25 μL of cDNA, 1.25 μL of pooled TaqMan Gene Expression Assays buffer (Life Technologies, ThermoFisher). The cDNA samples were subjected to pre-amplification following the temperature protocol - 2 min at 95 °C, followed by 12 cycles of 15 s at 95 °C and 4 min at 60 °C. The pre-amplified cDNA was diluted 5X by adding 20 μL of TEKNOVA. High-throughput real-time PCR was performed using the high-throughput BioMark HD System platform and the GE Dynamic Arrays (Fluidigm). Six microliters of sample master mix (SMM) consisted of 1.8 μL of 5X diluted pre-amplified cDNA, 0.3 μL of 20X GE Sample Loading Reagent (Fluidigm) and 3 μL of TaqMan Gene Expression PCR Master Mix (Life Technologies, ThermoFisher). Each 6 μL assay master mix (AMM) consisted of 3 μL of TaqMan Gene Expression assay 20×(Life Technologies) and 3 μL of 2X Assay Loading Reagent (Fluidigm). Five microliters of SMM and of AMM premixes were added to the dedicated wells. The samples and assays were mixed inside the chip using HX IFC controller (Fluidigm). Thermal conditions for qPCR were as follows: 30 min at 25 °C and 60 min at 70 °C for thermal mix; 2 min at 50 °C and 10 min at 95 °C for hot start; 40 cycles of 15 s at 95 °C and 1 min at 60 °C. Data were processed with an automatic threshold for each assay, with linear-derivative baseline correction using BioMark Real-Time PCR Analysis Software 4.0.1 (Fluidigm). The quality threshold was unchanged at the 0.65 default setting. Normalization was conducted using the mRNA expression of the *GAPDH* gene. Livak normalization provided the expression level in each sample with a transformation using the 2^ΔΔCT^ method^59^. 92 individuals had good quality expression data at baseline and 67 individuals had good quality expression data before and after the follow-up.

### Methylation data

Methylation levels on CpGs located in the seven YWHA genes were extracted from the methylomic data previously obtained for a subset of this cohort^4^. Briefly, for each individual, genomic DNA (500 ng) was extracted from whole blood and treated with sodium bisulfite using the EZ-96DNA Methylation KIT (Catalog No D5004, Zymo Research, Irvine, CA, USA) following the manufacturer’s standard protocol. Genome-wide DNA methylation was assessed using Illumina Infinium HumanMethylation450 BeadChip (Illumina, San Diego, CA, USA), which interrogates the DNA methylation profile of >485 000 CpG loci across the genome at single-nucleotide resolution. Illumina GenomeStudio software (Illumina) was used to extract signal intensities for each probe. For details about preprocessing and clean up steps, please refer to *Kebir et al*.^4^. Methylation and expression data were available for 25 patients (12 converters and 13 non converters) at both baseline and at the end of follow-up. A total of 122 CpGs were located in the promoter or the body of one of the seven YWHA genes. Methylation levels on these CpG were correlated with their corresponding gene expressions using Pearson’s test.

### Statistical analysis

Statistical analyses were performed using Python and R 3.5.1 software. Gene expression levels at baseline and longitudinal changes were standardized on the respective data of the non-converters using z-score. Logistic regressions (family: binomial), adjusted for participant age, sex, antipsychotic medication (chlorpromazine equivalent doses) were used to test the difference in standardized expression levels of YWHA genes at baseline (M0) and longitudinally between the two groups (converters and non-converters). A robust non-parametric significance (p) was computed through simulation-based random permutation analysis, whereby we randomly permuted the assignment of values to the groups, and repeated the statistical test 10,000 times. We then computed how many times a p-value was smaller or equal to the observed one. The reported P-value was calculated as the ratio of this number to the total number of tests done (10,000). In order to account for multiple comparison, p-value were adjusted using a false discovery rate (FDR) correction. Graphical representations were produced using Python and the ggplot2 R package^60^. Statistics and visualization of the correlation matrices were done with the R software, using the Hmisc and Corrplot packages^61–64^. Correlations between mRNA expression levels used Pearson’s correlation analysis. Correlations between mRNA expression levels and clinical scores used Spearman’s correlation analysis. PCA on the YWHA mRNA expression data was performed using the R package FactoMineR package^65^. The PCA determines the principal components of the correlated expression of the YWHA genes. Then, we compare the value of these principal components between the two clinical groups using a logistic regression model with age, sex and medication (chlorpromazine equivalent doses). In all cases, the differences were considered statistically significant when the corrected two-side p-values were lower than 0.05.

## Supplementary information

Supplementary information.

## Data Availability

The biological dataset is available from the corresponding author on reasonable request. Scripts for qPCR normalization are available on GitHub (https://github.com/jpouch/qPCR-Biomark). Statistical scripts are available from the corresponding author on reasonable request.
