# Supplementary material for: Dysregulation of peripheral expression of the YWHA genes during conversion to psychosis
Source: Sci Rep. 2020 Jun 17;10:9863. doi: 10.1038/s41598-020-66901-1 (PMC7299951; doi:10.1038/s41598-020-66901-1)
Supplement: Supplementary file 1 — Supplementary information. [file 41598_2020_66901_MOESM1_ESM.pdf]

Dysregulation of peripheral expression of the YWHA genes during conversion to psychosis

Fanny Demars[1], Oussama Kebir[1,2], Aude Marzo[1], Anton Iftimovici[1,3], Catherine Schramm[4], ICAAR Study Group, Marie-Odile Krebs [1,2,*], Boris Chaumette[1,2,5]

1) Institut de Psychiatrie et Neurosciences de Paris, INSERM UMR 1266, Laboratoire de Physiopathologie des Maladies Psychiatriques, Université de Paris, GDR3557-Institut de Psychiatrie, Paris, France
2) GHU Paris Psychiatrie et Neurosciences, Paris, France
3) NeuroSpin, Atomic Energy Commission, Unité d'Analyse et de Traitement de l'Information, Gif-sur-Yvette, France
4) CHU Sainte Justine, Université de Montréal, Montréal, Canada
5) Department of Psychiatry, McGill University, Montréal, Canada

*Corresponding author: Marie-Odile Krebs – mo.krebs@ghu-paris.fr – Pôle PEPIT – GHU Paris Psychiatrie et Neurosciences - 7 rue Cabanis – 75014 Paris (France)

Contributors of the ICAAR Study Group:

Isabelle Amado[2], Julie Bourgin[2], Claire Daban Huard[2], Célia Jantac Mam-Lam-Fook[2], Marion Plaze[2], Fabrice Rivollier[2]

Supplementary Table 1: Correlation matrix between baseline expression levels of YWHA genes and clinical scores at the end of the follow-up

	CGI	SOFAS	BPRS	PANSS POSITIVE	PANSS NEGATIVE	PANSS GENERAL	PANSS TOTALE	MADRS	YMRS	CAARMS score
YWHAB	0.232	-0.202	0.148	0.009	**0.374 ****	0.232	0.247	-0.039	-0.213	0.205
YWHAE	0.211	**-0.375 ****	0.062	0.079	**0.294 ***	0.101	0.178	0.177	-0.011	0.239
YWHAG	0.098	-0.173	0.145	0.23	0.073	0.066	0.152	-0.032	0.012	**0.311 ***
YWHAH	-0.048	0.029	0.008	0.095	-0.091	-0.009	-0.004	-0.114	-0.113	0.041
YWHAQ	0.171	**-0.33 ***	**0.262 ***	0.117	**0.369 ****	0.111	**0.265 ***	-0.065	0.073	**0.361 ****
YWHAS	-0.169	0.031	-0.04	-0.224	0.237	-0.006	0.027	**-0.269 ***	-0.127	-0.031
YWHAZ	0.118	-0.123	0.164	0.203	0.147	0.075	0.154	0.098	-0.09	0.259
First Dimension of the PCA	0,155	-0,255	0,151	0,111	0,266	0,01	0,191	-0,058	-0,051	**0,341 ***

Each cell indicates the Spearman correlation coefficient between clinical scores at the end of follow-up and baseline YWHA mRNA expression levels irrespective of the clinical status of the participant. Coefficients with p-values <0.05 are in bold (* p ≤ 0.05 ** p ≤ 0.01 ***p ≤ 0.001)

Supplementary Table 2: Correlation matrix between the expression levels of the different YWHA genes

	**YWHAB**	**YWHAE**	**YWHAG**	**YWHAH**	**YWHAQ**	**YWHAS**	**YWHAZ**
**YWHAB**	1						
**YWHAE**	**0.34*****	1					
**YWHAG**	**0.23****	**0.40*****	1				
**YWHAH**	-0.01	**0.33*****	**0.72*****	1			
**YWHAQ**	**0.47*****	**0.35*****	**0.24****	0.02	1		
**YWHAS**	**0.28****	**0.17***	0.05	-0.03	**0.34*****	1	
**YWHAZ**	**0.17***	**0.48*****	**0.48*****	**0.50*****	**0.29*****	0.06	1

Each cell indicates the Pearson correlation coefficient between the mRNA expression levels of different YWHA genes for all available samples (baseline and end of follow-up). P-values <0.05 are in bold (* p ≤ 0.05 ** p ≤ 0.01 ***p ≤ 0.001)

Supplementary Table 3: Significant Pearson's correlations between CpG methylation and expression levels of YWHA genes

Gene	CpG	Genomic Coordinate	Chromosome	CpG location	Correlation coefficient	P-value
YWHAG	cg03707599	75956931	7	intragenic	0,457	0,0023
YWHAG	cg10167463	75959203	7	intragenic	0,427	0,0048
YWHAG	cg10738119	75957040	7	intragenic	0,316	0,041
YWHAG	cg17100201	75958978	7	intragenic	0,316	0,041
YWHAG	cg26162326	75957061	7	intragenic	0,357	0,020
YWHAZ	cg03720887	101963845	8	intragenic, promoter	-0,344	0,027
YWHAZ	cg04962480	101962118	8	intragenic	-0,370	0,017

Only significant results with P-values <0.05 are shown.

Supplementary Table 4: Studies used as reference for the computation of chlorpromazine equivalents

Typical antipsychotic	Reference study
Klorprotixene	(Kroken et al., 2009; Leucht et al., 2014) [1,2]
Levomepromazine	(Kroken et al., 2009) [1]
Triflupromazine	(Davis, 1975) [3]
Thioridazine	(Andreasen et al., 2010) [4]
Dixyrazine	(Kroken et al., 2009) [1]
Prochlorperazine	(Davis, 1975; Woods, 2005) [3,5]
Perphenazine	(Andreasen et al., 2010) [4]
Perphenazine decanoate	(Kroken et al., 2009) [1]
Zuclopenthixol	(Kroken et al., 2009) [1]
Zuclopenthixol decanoate	(Kroken et al., 2009) [1]
Flupenthixol	(Kroken et al., 2009) [1]
Flupenthixol decanoate	(Kroken et al., 2009) [1]
Fluphenazine	(Andreasen et al., 2010) [4]
Fluphenazine decanoate	(Andreasen et al., 2010) [4]
Trifluoperazine	(Andreasen et al., 2010) [4]
Acetophenazine	(Davis, 1975; Leucht et al., 2014) [2,3]

Carphenazine	(Davis, 1975; Leucht et al., 2014)	[2,3]
Butaperazine	(Davis, 1975; Leucht et al., 2014)	[2,3]
Mesoridazine	(Davis, 1975; Leucht et al., 2014)	[2,3]
Piperacetazine	(Davis, 1975; Leucht et al., 2014)	[2,3]
Haloperidol	(Andreasen et al., 2010)	[4]
Haloperidol decanoate	(Andreasen et al., 2010)	[4]
Chlorprothixene	(Davis, 1975)	[3]
Thiothixene	(Andreasen et al., 2010)	[4]
Molindone	(Woods, 2005)	[5]
Prochlorperazine	(Leucht et al., 2014; Woods, 2005)	[2,5]
Atypical antipsychotics		
Risperidone	(Andreasen et al., 2010)	[4]
Risperidone action prolongée	(Kroken et al., 2009; Woods, 2005)	[1,5]
Olanzapine	(Andreasen et al., 2010)	[4]
Quetiapine	(Andreasen et al., 2010)	[4]
Ziprasidone	(Andreasen et al., 2010)	[4]
Aripiprazole	(Andreasen et al., 2010)	[4]
clozapine	(Andreasen et al., 2010)	[4]
Asenapine	(Leucht et al., 2014; Woods, 2005)	[2,5]
Iloperidone	(Leucht et al., 2014; Woods, 2005)	[2,5]
Lurasidone	(Leucht et al., 2014; Woods, 2005)	[2,5]
Paliperidone	(Leucht et al., 2014; Woods, 2005)	[2,5]
Sertindole	(Kroken et al., 2009; Leucht et al., 2014)	[1,2]
Amisulpride	(Bazire, 2007)	[6]
Sulpride	(Bazire, 2007)	[6]

Supplementary Figure 1: Random permutations analysis on the logistic regressions used to compare baseline expression of YWHA genes between converters and non-converters

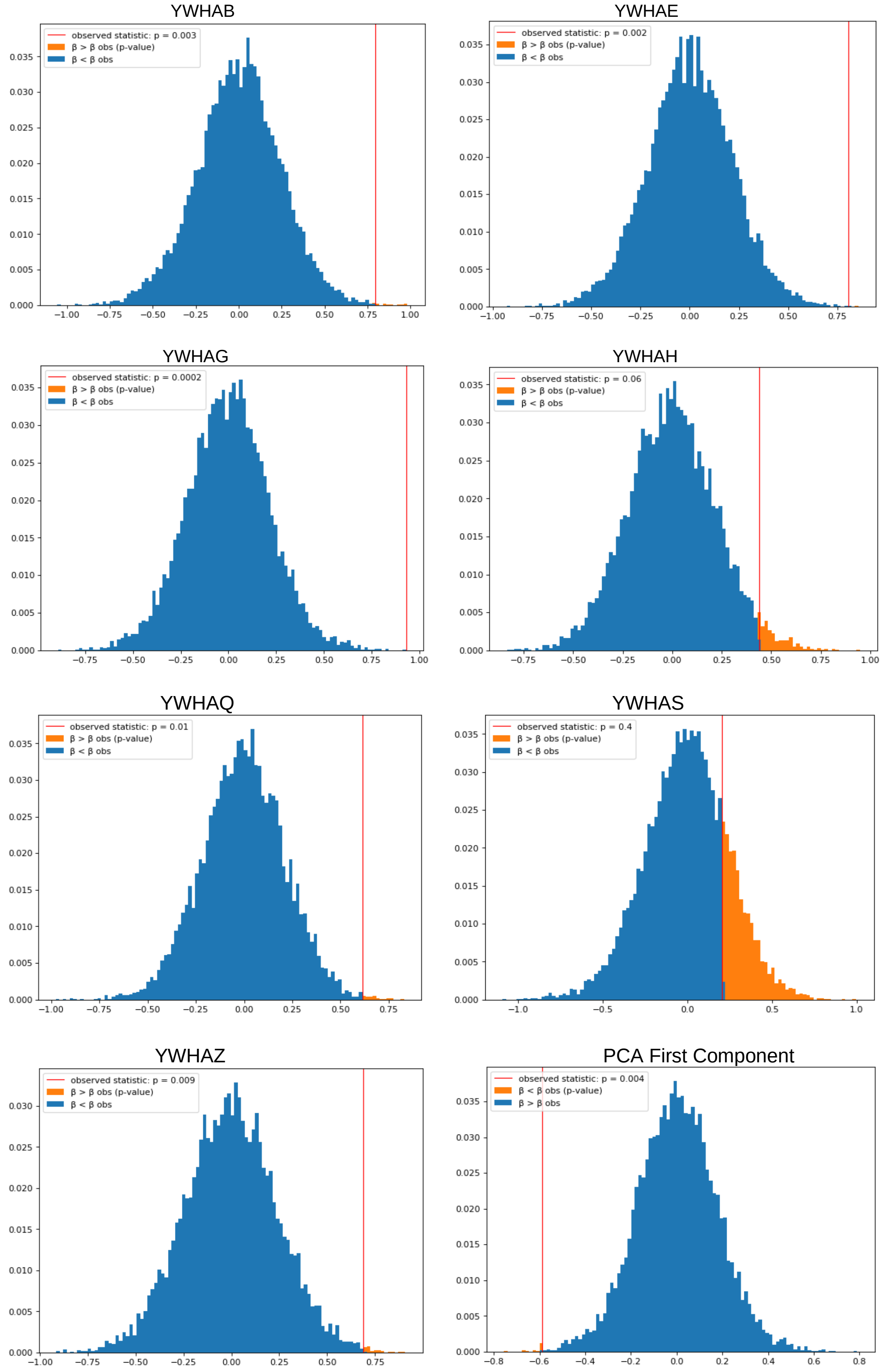

Supplementary Figure 2: Random permutations analysis on the logistic regressions used to compare longitudinal changes in expression of YWHA genes between converters and non-converters

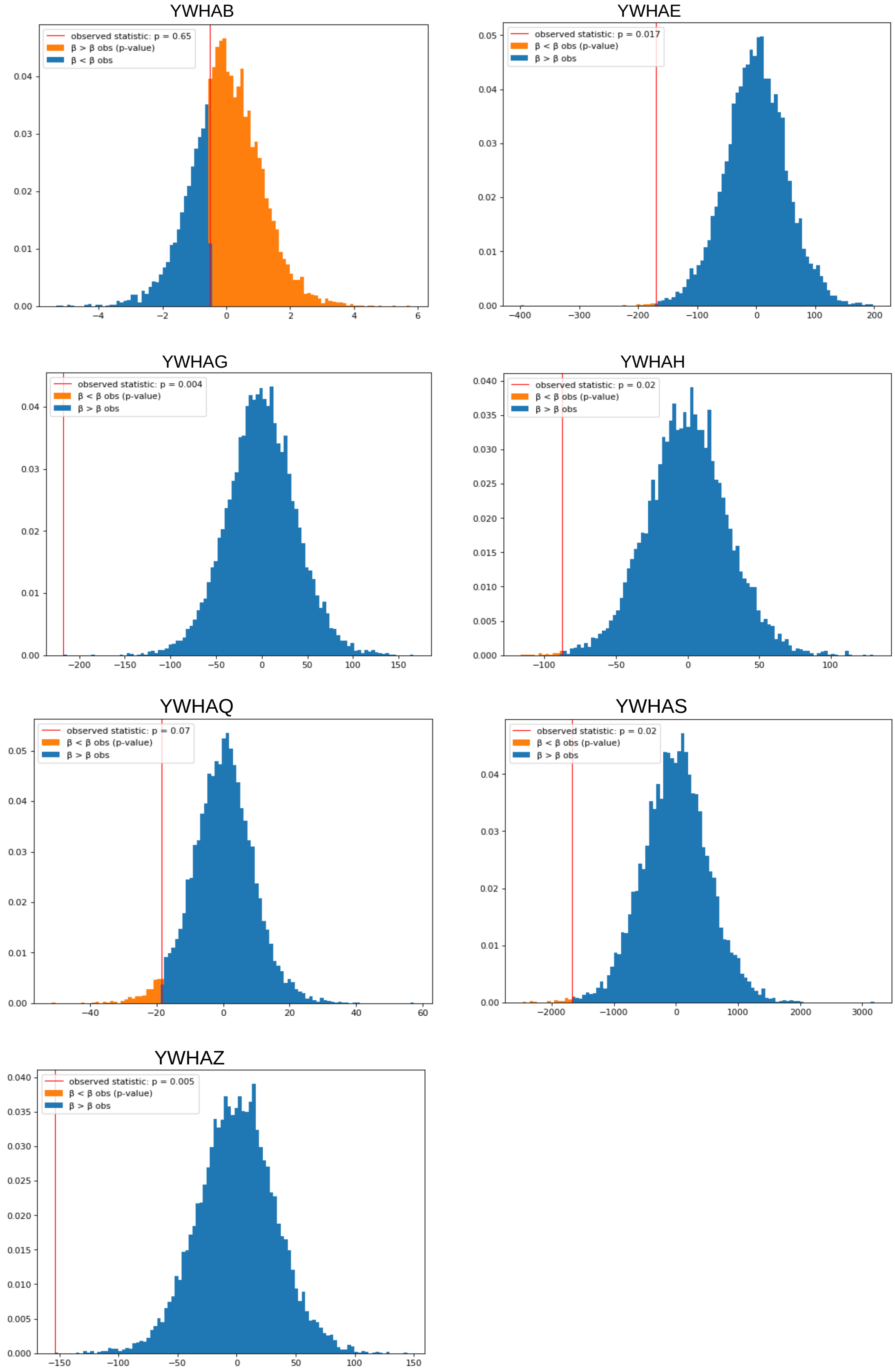

Supplementary Figure 3: Correlation plot between the expression levels of the different YWHA genes

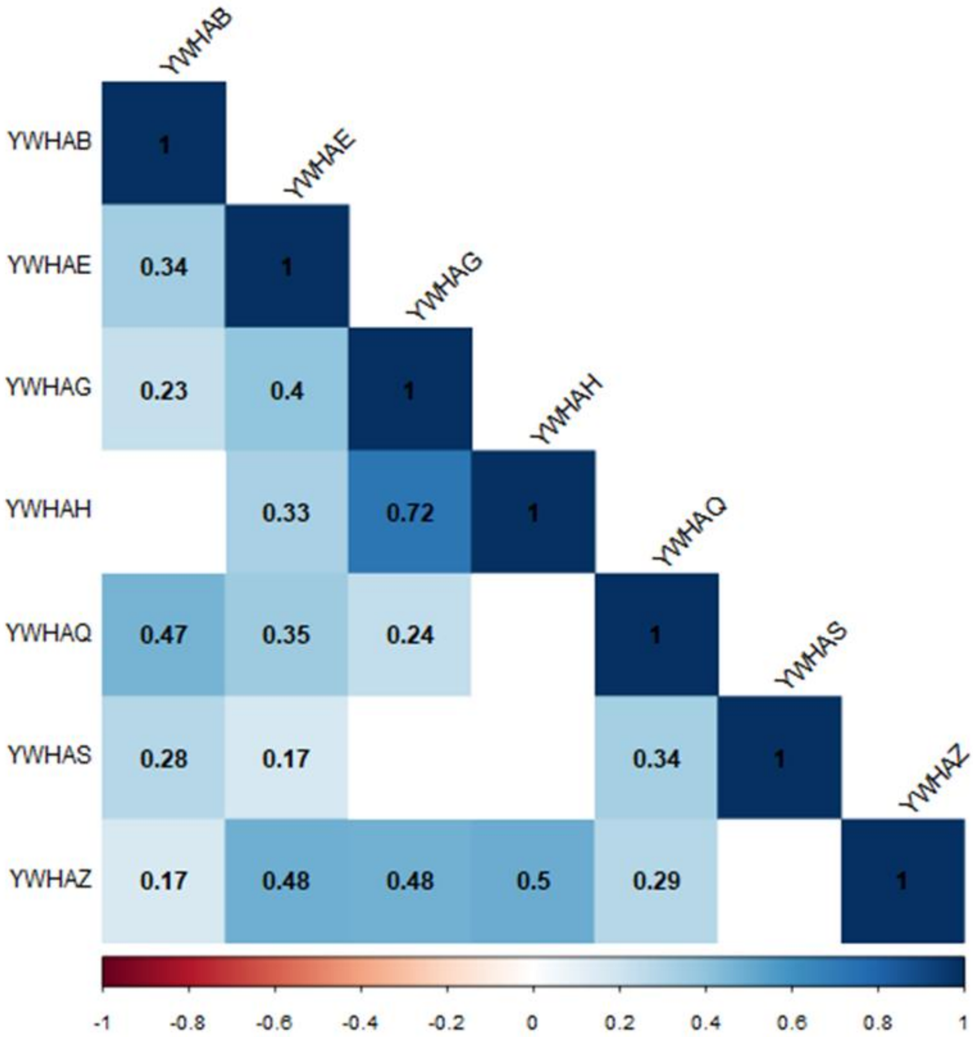

Color intensity is proportional to the value of Pearson correlation coefficient that is indicated inside of each cell. The positive correlations are displayed in blue and negative correlations in red (the color code for the correlation values is presented below the matrix). The non-significant correlation coefficients (p>0.05) are not displayed (blank cases in the matrix).

Supplementary references :

1. Kroken, R. A., Johnsen, E., Ruud, T., Wentzel-Larsen, T. & Jørgensen, H. A. Treatment of schizophrenia with antipsychotics in Norwegian emergency wards, a cross-sectional national study. BMC Psychiatry 9, 24 (2009).

2. Leucht, S. et al. Dose equivalents for second-generation antipsychotics: the minimum effective dose method. Schizophr. Bull. 40, 314–326 (2014).

3. Davis, J. M. Dose equivalence of the antipsychotic drugs. in Catecholamines and Schizophrenia 65–73 (Elsevier, 1975).

4. Andreasen, N. C., Pressler, M., Nopoulos, P., Miller, D. & Ho, B.-C. Antipsychotic dose equivalents and dose-years: a standardized method for comparing exposure to different drugs. Biol. Psychiatry 67, 255–262 (2010).

5. Woods, S. W. Calculation of CPZ Equivalents. at <www.scottwilliamwoods.com/files/Equivtext.doc> (2005).

6. Bazire, S. Psychotropic Drug Directory, Maudsley Guideline. (2007).
